# Supplementary material for: T7Max transcription system
Source: J Biol Eng. 2023 Jan 23;17:4. doi: 10.1186/s13036-023-00323-1 (PMC9872363; doi:10.1186/s13036-023-00323-1)
Supplement: Supplementary file 1 — Additional file 1: Figure S1. The original uncropped gels for data presented on Fig. 1. Sybr stained gel. [file 13036_2023_323_MOESM1_ESM.docx]

**Figure S1**


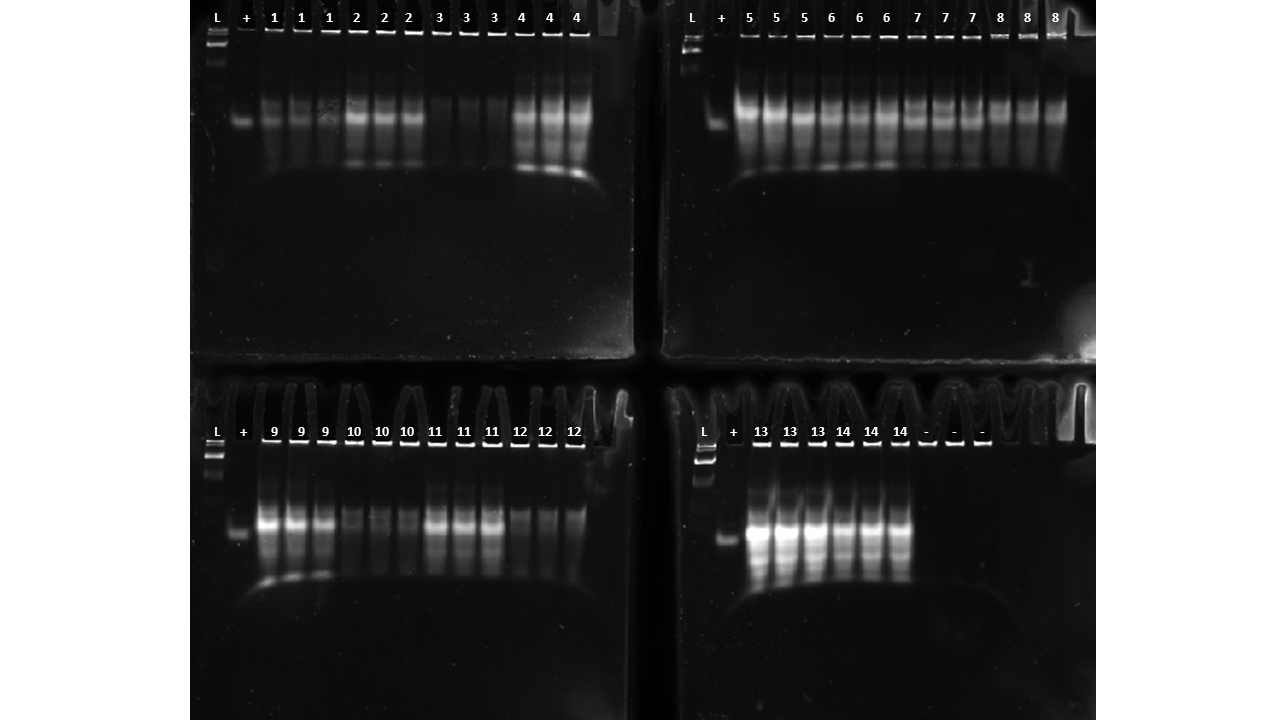


**Figure S1**. The original uncropped gels for data presented on Figure 1. Sybr stained gel, samples are:

| Sample ID | Sense strand |
| --- | --- |
| L | Low Range ssRNA Ladder (N0365S) |
| Control broccoli | 10 pmol broccoli |
| 1 | uhlenbeck minimal promoter |
| 2 | unlenbeck GG promoter |
| 3 | T7c62 promoter |
| 4 | uhlenbeck 600 promoter |
| 5 | uhlenbeck 500 promoter |
| 6 | uhlenbeck 400 promoter |
| 7 | uhlenbeck 325 promoter |
| 8 | uhlenbeck 230 promoter |
| 9 | uhlenbeck 117 promoter |
| 10 | uhlenbeck 73 promoter |
| 11 | uhlenbeck 45 promoter |
| 12 | uhlenbeck 15 promoter |
| 13 | NASBA promoter |
| 14 | T7Max Promoter |
| - | No Template Control |
