# Supplementary figures and images for: T7Max transcription system
Source: J Biol Eng. 2023 Jan 23;17:4. doi: 10.1186/s13036-023-00323-1 (PMC9872363; doi:10.1186/s13036-023-00323-1)

**Figure S3**


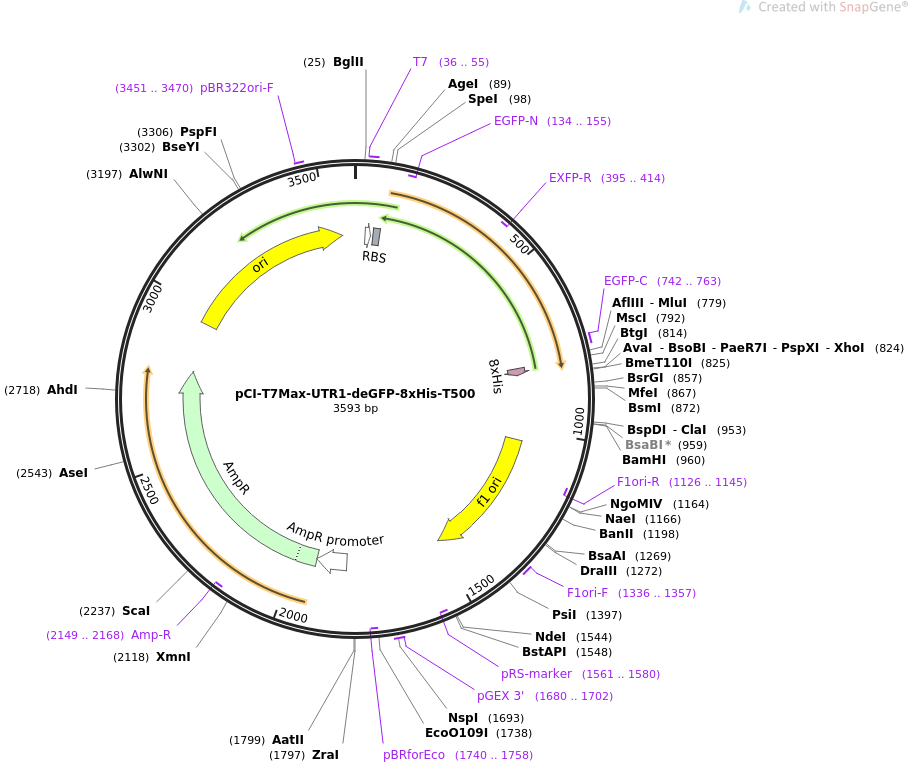


**Figure S3**. Map of plasmid used in the TxTl experiments, pCI-T7Max-UTR1-deGFP-8xHis-T500.

Supplement: Supplementary file 3 — Additional file 3: Figure S3. Map of plasmid used in the TxTl experiments, pCI-T7Max-UTR1-deGFP-8xHis-T500. [file 13036_2023_323_MOESM3_ESM.docx]

**Figure S4**


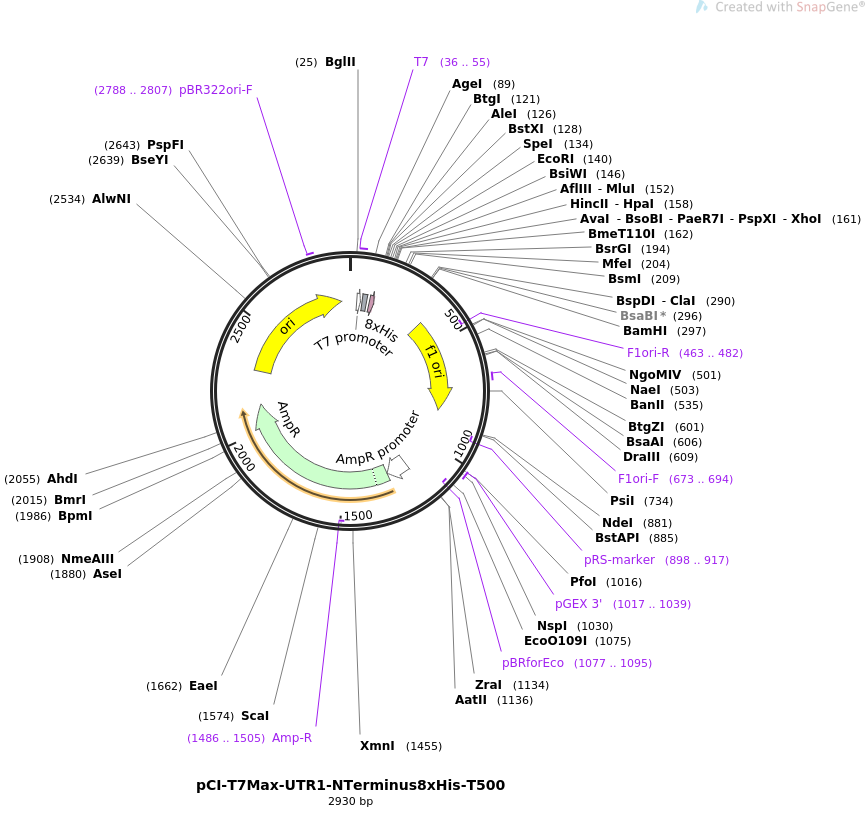


**Figure S4**. Map of plasmid used in the TxTl experiments, pCI-T7Max-UTR1-NTerminus8xHis-T500.

Supplement: Supplementary file 4 — Additional file 4: Figure S4. Map of plasmid used in the TxTl experiments, pCI-T7Max-UTR1-NTerminus8xHis-T500. [file 13036_2023_323_MOESM4_ESM.docx]

**Figure S5**


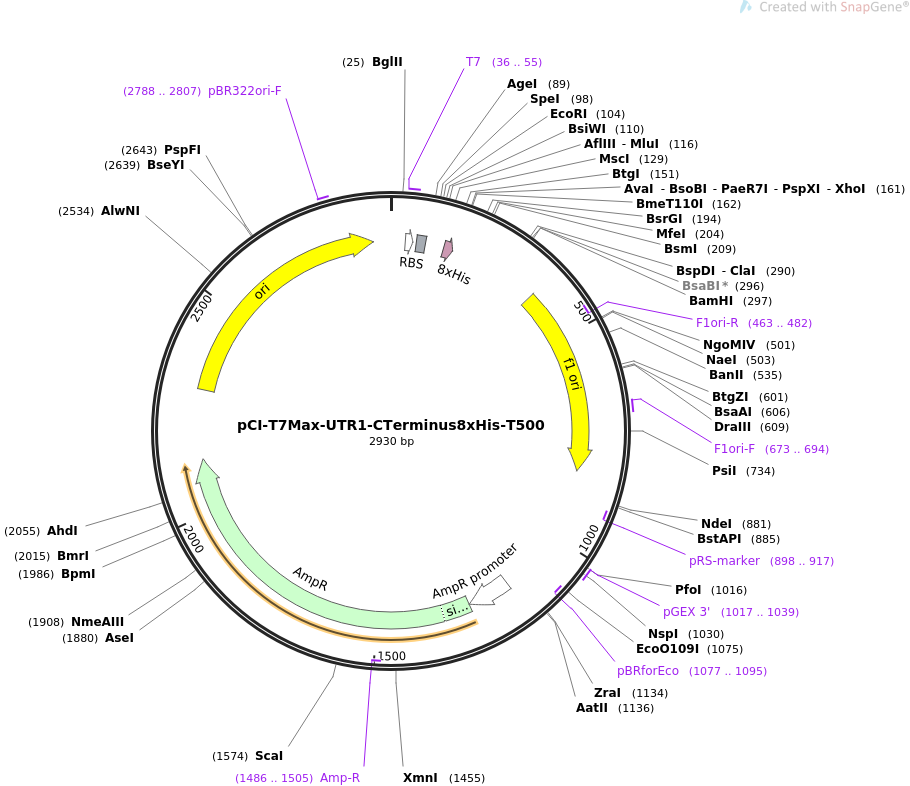


**Figure S5**. Map of plasmid used in the TxTl experiments, pCI-T7Max-UTR1-CTerminus8xHis-T500.

Supplement: Supplementary file 5 — Additional file 5: Figure S5. Map of plasmid used in the TxTl experiments, pCI-T7Max-UTR1-CTerminus8xHis-T500. [file 13036_2023_323_MOESM5_ESM.docx]
