# Supplementary material for: T7Max transcription system
Source: J Biol Eng. 2023 Jan 23;17:4. doi: 10.1186/s13036-023-00323-1 (PMC9872363; doi:10.1186/s13036-023-00323-1)
Supplement: Supplementary file 6 — Additional file 6: Figure S6. The full uncropped Western Blot image of GFP expression comparison between T7 and T7Max. p1686 is T7 and p2008 is T7Max promoter. [file 13036_2023_323_MOESM6_ESM.docx]

**Figure S6**


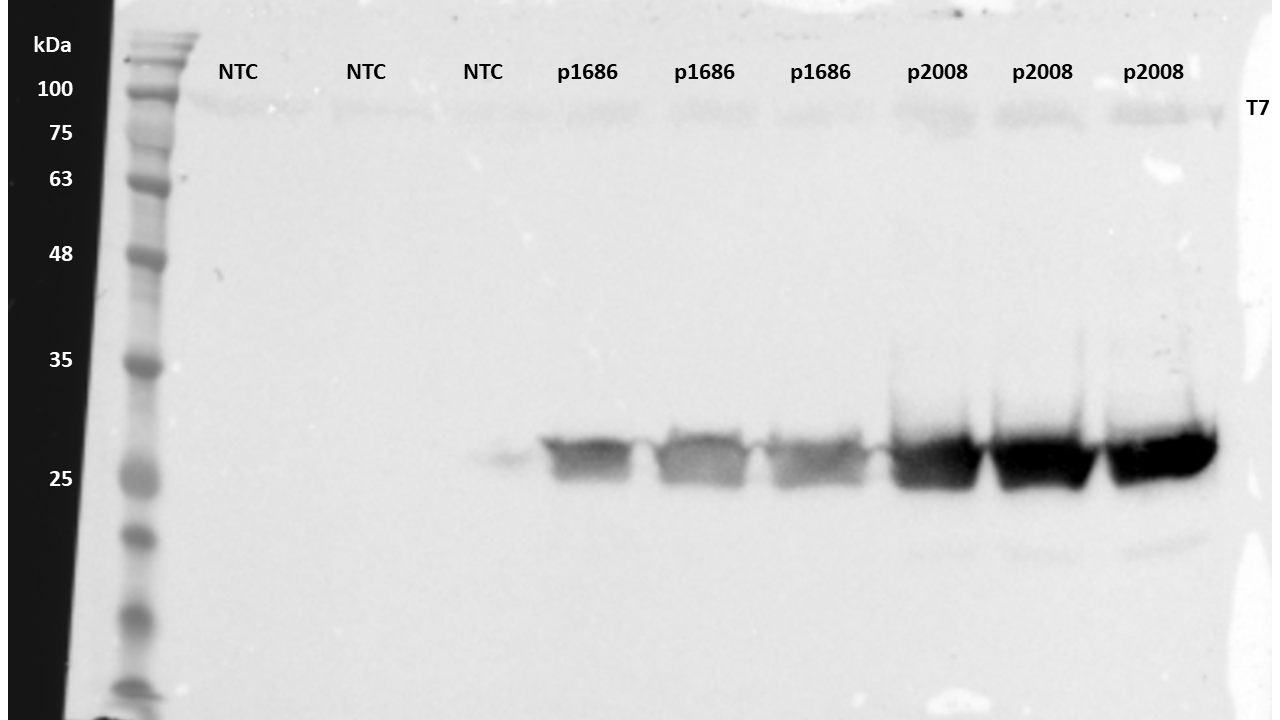


**Figure S6**. The full uncropped Western Blot image of GFP expression comparison between T7 and T7Max. p1686 is T7 and p2008 is T7Max promoter.
