# Supplementary material for: T7Max transcription system
Source: J Biol Eng. 2023 Jan 23;17:4. doi: 10.1186/s13036-023-00323-1 (PMC9872363; doi:10.1186/s13036-023-00323-1)
Supplement: Supplementary file 7 — Additional file 7: Figure S7. Western blots used to quantify protein expression for different proteins using T7 vs T7Max promoter. The ladder is BLUEstain Protein Ladder (Goldbio). [file 13036_2023_323_MOESM7_ESM.docx]

**Figure S7**


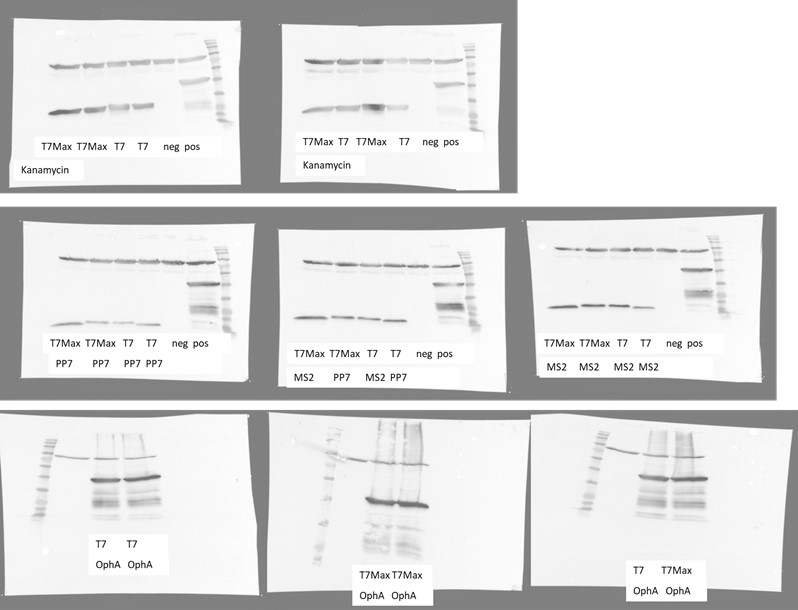


**Figure S7**. Western blots used to quantify protein expression for different proteins using T7 vs T7Max promoter. The ladder is BLUEstain Protein Ladder (Goldbio).
