# Supplementary material for: T7Max transcription system
Source: J Biol Eng. 2023 Jan 23;17:4. doi: 10.1186/s13036-023-00323-1 (PMC9872363; doi:10.1186/s13036-023-00323-1)
Supplement: Supplementary file 8 — Additional file 8: Figure S8. GFP fluorescence data for expression of proteins in different cell-free extracts, before normalizing T7 value to 100 (as shown on Fig. 7). [file 13036_2023_323_MOESM8_ESM.docx]

**Figure S8**


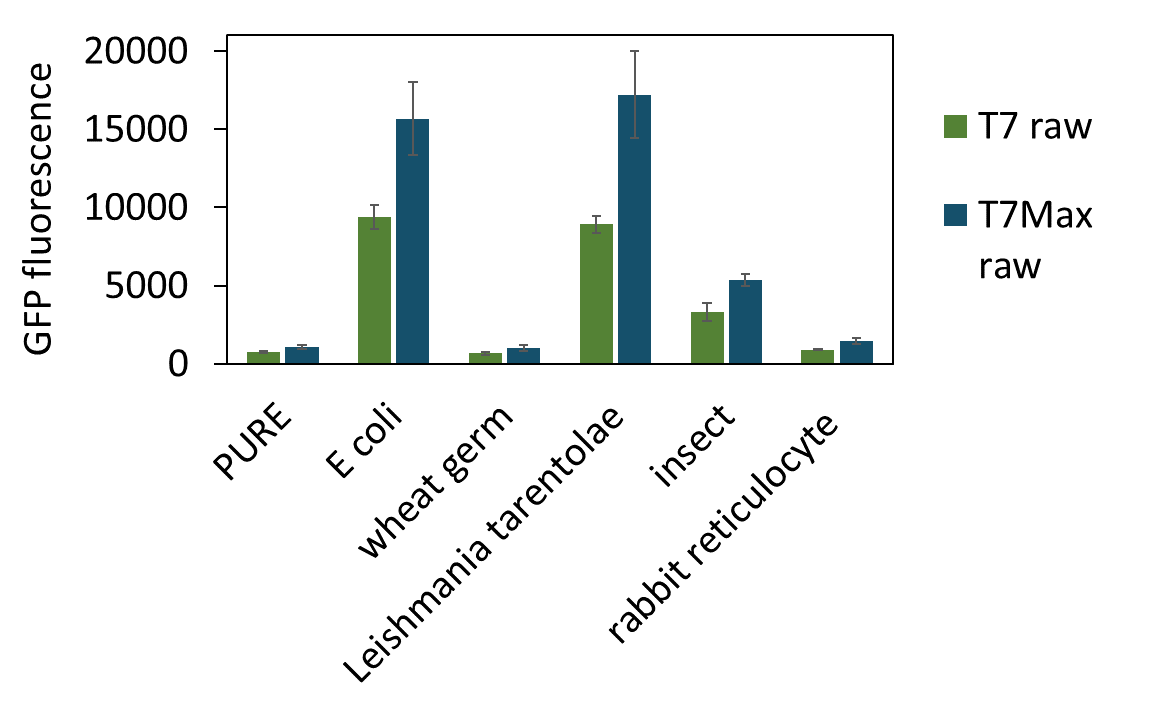


**Figure S8**. GFP fluorescence data for expression of proteins in different cell-free extracts, before normalizing T7 value to 100 (as shown on **figure 7**).
