# Supplementary material for: T7Max transcription system
Source: J Biol Eng. 2023 Jan 23;17:4. doi: 10.1186/s13036-023-00323-1 (PMC9872363; doi:10.1186/s13036-023-00323-1)
Supplement: Supplementary file 9 — Additional file 9: Table S1. Complete sequences used in the promoter testing experiments. The aptamer transcription templates had no terminators, so all transcriptions were run-off terminated. Promoter sequence is underlined, the aptamer sequence is in italics and protein coding sequence is in bold. [file 13036_2023_323_MOESM9_ESM.docx]

# Table S1

Complete sequences used in the promoter testing experiments. The aptamer transcription templates had no terminators, so all transcriptions were run-off terminated.

Promoter sequence is underlined, the aptamer sequence is in *italics* and protein coding sequence is in **bold**.

| Oligo name (based on promoter name) | Sequence |
| --- | --- |
| NASBA Promoter | AATTTAATACGACTCACTATAGGGA*GAGACGGTCGGGTCCAGATATTC* |
| T7Max | AATTCTAATACGACTCACTATAGGGA*GAGACGGTCGGGTCCAGATATTC* |
| Uhlenbeck 15 | TAATACGACTCACTATACGATCA*GAGACGGTCGGGTCCAGATATTC* |
| Uhlenbeck 45 | TAATACGACTCACTATAGGACAT*GAGACGGTCGGGTCCAGATATTC* |
| Uhlenbeck Minimal | TAATACGACTCACTATA*GAGACGGTCGGGTCCAGATATTC* |
| T7wt | TAATACGACTCACTATAGG*GAGACGGTCGGGTCCAGATATTC* |
| T7c62 | TAATACGACTCACAATCGCGGAG*GAGACGGTCGGGTCCAGATATTC* |
| Uhlenbeck 600 | TAATACGACTCACTATAGGGATC*GAGACGGTCGGGTCCAGATATTC* |
| Uhlenbeck 500 | TAATACGACTCACTATAGGGAGA*GAGACGGTCGGGTCCAGATATTC* |
| Uhlenbeck 400 | TAATACGACTCACTATAGGGACT*GAGACGGTCGGGTCCAGATATTC* |
| Uhlenbeck 325 | TAATACGACTCACTATAGGGCTC*GAGACGGTCGGGTCCAGATATTC* |
| Uhlenbeck 230 | TAATACGACTCACTATAGAGACT*GAGACGGTCGGGTCCAGATATTC* |
| Uhlenbeck 117 | TAATACGACTCACTATAGGGAAG*GAGACGGTCGGGTCCAGATATTC* |
| Uhlenbeck 73 | TAATACGACTCACTATAGCATCA*GAGACGGTCGGGTCCAGATATTC* |
| Uhlenbeck 117 – GFP template | TAATACGACTCACTATAGGGAAGAATAATTTTGTTTAACTTTAAGAAGGAGATATACCGGTATGACTAGT**ATGGAGCTTTTCACTGGCGTTGTTCCCATCCTGGTCGAGCTGGACGGCGACGTAAACGGCCACAAGTTCAGCGTGTCCGGCGAGGGCGAGGGCGATGCCACCTACGGCAAGCTGACCCTGAAGTTCATCTGCACCACCGGCAAGCTGCCCGTGCCCTGGCCCACCCTCGTGACCACCCTGACCTACGGCGTGCAGTGCTTCAGCCGCTACCCCGACCACATGAAGCAGCACGACTTCTTCAAGTCCGCCATGCCCGAAGGCTACGTCCAGGAGCGCACCATCTTCTTCAAGGACGACGGCAACTACAAGACCCGCGCCGAGGTGAAGTTCGAGGGCGACACCCTGGTGAACCGCATCGAGCTGAAGGGCATCGACTTCAAGGAGGACGGCAACATCCTGGGGCACAAGCTGGAGTACAACTACAACAGCCACAACGTCTATATCATGGCCGACAAGCAGAAGAACGGCATCAAGGTGAACTTCAAGATCCGCCACAACATCGAGGACGGCAGCGTGCAGCTCGCCGACCACTACCAGCAGAACACCCCCATCGGCGACGGCCCCGTGCTGCTGCCCGACAACCACTACCTGAGCACCCAGTCCGCCCTGAGCAAAGACCCCAACGAGAAGCGCGATCACATGGTCCTGCTGGAGTTCGTGACCGCCGCCGGGATCACGCGTGGAGGTGGCCATCACCATCATCACCATCACCACGGATAA**CTCGAGCAAAGCCCGCCGAAAGGCGGGCTTTTCTGT |
| T7Max – GFP template | AATTCTAATACGACTCACTATAGGGAAATAATTTTGTTTAACTTTAAGAAGGAGATATACCGGTATGACTAGT**ATGGAGCTTTTCACTGGCGTTGTTCCCATCCTGGTCGAGCTGGACGGCGACGTAAACGGCCACAAGTTCAGCGTGTCCGGCGAGGGCGAGGGCGATGCCACCTACGGCAAGCTGACCCTGAAGTTCATCTGCACCACCGGCAAGCTGCCCGTGCCCTGGCCCACCCTCGTGACCACCCTGACCTACGGCGTGCAGTGCTTCAGCCGCTACCCCGACCACATGAAGCAGCACGACTTCTTCAAGTCCGCCATGCCCGAAGGCTACGTCCAGGAGCGCACCATCTTCTTCAAGGACGACGGCAACTACAAGACCCGCGCCGAGGTGAAGTTCGAGGGCGACACCCTGGTGAACCGCATCGAGCTGAAGGGCATCGACTTCAAGGAGGACGGCAACATCCTGGGGCACAAGCTGGAGTACAACTACAACAGCCACAACGTCTATATCATGGCCGACAAGCAGAAGAACGGCATCAAGGTGAACTTCAAGATCCGCCACAACATCGAGGACGGCAGCGTGCAGCTCGCCGACCACTACCAGCAGAACACCCCCATCGGCGACGGCCCCGTGCTGCTGCCCGACAACCACTACCTGAGCACCCAGTCCGCCCTGAGCAAAGACCCCAACGAGAAGCGCGATCACATGGTCCTGCTGGAGTTCGTGACCGCCGCCGGGATCACGCGTGGAGGTGGCCATCACCATCATCACCATCACCACGGATAA**CTCGAGCAAAGCCCGCCGAAAGGCGGGCTTTTCTGT |
| regular T7 – GFP template | TAATACGACTCACTATAGGAATAATTTTGTTTAACTTTAAGAAGGAGATATACCGGTATGACTAGT**ATGGAGCTTTTCACTGGCGTTGTTCCCATCCTGGTCGAGCTGGACGGCGACGTAAACGGCCACAAGTTCAGCGTGTCCGGCGAGGGCGAGGGCGATGCCACCTACGGCAAGCTGACCCTGAAGTTCATCTGCACCACCGGCAAGCTGCCCGTGCCCTGGCCCACCCTCGTGACCACCCTGACCTACGGCGTGCAGTGCTTCAGCCGCTACCCCGACCACATGAAGCAGCACGACTTCTTCAAGTCCGCCATGCCCGAAGGCTACGTCCAGGAGCGCACCATCTTCTTCAAGGACGACGGCAACTACAAGACCCGCGCCGAGGTGAAGTTCGAGGGCGACACCCTGGTGAACCGCATCGAGCTGAAGGGCATCGACTTCAAGGAGGACGGCAACATCCTGGGGCACAAGCTGGAGTACAACTACAACAGCCACAACGTCTATATCATGGCCGACAAGCAGAAGAACGGCATCAAGGTGAACTTCAAGATCCGCCACAACATCGAGGACGGCAGCGTGCAGCTCGCCGACCACTACCAGCAGAACACCCCCATCGGCGACGGCCCCGTGCTGCTGCCCGACAACCACTACCTGAGCACCCAGTCCGCCCTGAGCAAAGACCCCAACGAGAAGCGCGATCACATGGTCCTGCTGGAGTTCGTGACCGCCGCCGGGATCACGCGTGGAGGTGGCCATCACCATCATCACCATCACCACGGATAA**CTCGAGCAAAGCCCGCCGAAAGGCGGGCTTTTCTGT |
| PP7 monomer | **ATGGAGCATCATCATCATCATCATCATCATGGACTCGCCTCTAAAACGATCGTTCTTTCAGTGGGCGAAGCCACCCGCACTCTTACTGAGATCCAATCTACCGCTGATAGGCAGATTTTTGAGGAAAAAGTCGGCCCCCTGGTGGGACGACTTCGGCTCACCGCATCCTTGAGGCAGAATGGCGCTAAGACTGCTTACAGAGTGAACCTTAAGCTGGACCAAGCAGATGTGGTTGACAGTGGCCTGCCCAAAGTTAGATACACCCAGGTCTGGAGTCACGACGTTACCATCGTCGCCAACAGCACTGAGGCCTCCCGGAAGTCCTTGTATGATCTGACCAAATCCCTTGTGGCCACAAGTCAGGTCGAAGACCTTGTGGTTAACCTTGTACCTCTGGGACGCTAA** |
| OphA | **ATGACTTCCACTCAGACCAAAGCTGGCTCACTCACCATCGTCGGTACCGGTATCGAGAGTATCGGACAAATGACGCTTCAGGCGTTGTCCTACATCGAAGCCGCCGCGAAGGTTTTCTACTGCGTCATCGACCCCGCCACTGAGGCATTCATCCTCACCAAGAACAAGAACTGCGTTGACTTGTATCAGTATTACGACAATGGCAAGTCCAGATTGAACACTTACACCCAAATGTCAGAGCTCATGGTCAGGGAAGTCCGCAAGGGCCTCGATGTCGTGGGCGTCTTCTACGGCCACCCAGGAGTGTTCGTGAACCCGTCTCACCGAGCTCTGGCTATCGCCAAGAGTGAAGGCTACCGAGCGAGGATGCTTCCAGGCGTGTCTGCGGAAGATTGTCTCTTCGCGGACTTGTGCATTGATCCTTCGAACCCGGGTTGCCTGACCTACGAGGCATCGGATTTCCTGATCAGGGATCGCCCGGTCAGCATCCACAGTCACTTGGTCCTGTTCCAAGTCGGATGCGTCGGTATCGCCGACTTCAACTTCACTGGATTCGACAACAACAAATTCGGCGTTCTCGTCGACCGTCTCGAGCAAGAATACGGCGCCGAGCACCCTGTCGTCCATTACATCGCAGCTATGATGCCACACCAAGACCCAGTCACCGATAAATACACCGTCGCGCAGCTCCGTGAGCCCGAGATCGCGAAGCGTGTTGGCGGTGTCTCGACTTTCTACATCCCTCCCAAGGCCAGGAAAGCATCGAACTTGGACATCATAAGGCGCCTAGAGCTCTTGCCTGCTGGGCAAGTTCCCGACAAGAAAGCGCGTATTTACCCGGCCAACCAGTGGGAGCCCGATGTTCCCGAAGTCGAACCCTACAGACCATCTGACCAGGCTGCCATCGCTCAGTTGGCTGACCACGCTCCTCCTGAGCAATATCAACCTCTTGCTACTTCGAAAGCCATGTCTGATGTTATGACGAAGTTGGCTTTGGATCCCAAGGCACTCGCCGACTACAAAGCTGATCACCGCGCCTTTGCTCAATCTGTCCCCGACTTGACGCCTCAGGAGCGTGCGGCTTTGGAGCTCGGTGATTCGTGGGCTATTCGTTGCGCGATGAAGAATATGCCCTCGTCGCTCTTGGACGCTGCTCGTGAATCCGGCGAAGAGGCATCCCAAAACGGTTTCCCATGGGTCATCGTCGTTGGTGTTATCGGTGTCATCGGATCAGTCATGAGCACGGAATAA** |
| Kanamycin resistance | **ATGAGCCATATTCAACGGGAAACGTCGAGGCCGCGATTAAATTCCAACATGGATGCTGATTTATATGGGTATAAATGGGCTCGCGATAATGTCGGGCAATCAGGTGCGACAATCTATCGCTTGTATGGGAAGCCCGATGCGCCAGAGTTGTTTCTGAAACATGGCAAAGGTAGCGTTGCCAATGATGTTACAGATGAGATGGTCAGACTAAACTGGCTGACGGAATTTATGCCTCTTCCGACCATCAAGCATTTTATCCGTACTCCTGATGATGCATGGTTACTCACCACTGCGATCCCCGGAAAAACAGCATTCCAGGTATTAGAAGAATATCCTGATTCAGGTGAAAATATTGTTGATGCGCTGGCAGTGTTCCTGCGCCGGTTGCATTCGATTCCTGTTTGTAATTGTCCTTTTAACAGCGATCGCGTATTTCGTCTCGCTCAGGCGCAATCACGAATGAATAACGGTTTGGTTGATGCGAGTGATTTTGATGACGAGCGTAATGGCTGGCCTGTTGAACAAGTCTGGAAAGAAATGCATAAACTTTTGCCATTCTCACCGGATTCAGTCGTCACTCATGGTGATTTCTCACTTGATAACCTTATTTTTGACGAGGGGAAATTAATAGGTTGTATTGATGTTGGACGAGTCGGAATCGCAGACCGATACCAGGATCTTGCCATCCTATGGAACTGCCTCGGTGAGTTTTCTCCTTCATTACAGAAACGGCTTTTTCAAAAATATGGTATTGATAATCCTGATATGAATAAATTGCAGTTTCATTTGATGCTCGATGAGTTTTTCACGCGTGGAGGTGGCCATCACCATCATCACCATCACCACGGATAA** |
| MS2 monomer | **ATGGAGCATCATCATCATCATCATCATCATGGAATGGCCTCCAACTTTACGCAATTTGTGCTTGTCGATAACGGGGGCACAGGGGATGTGACTGTGGCTCCTTCCAACTTCGCAAACGGCATAGCAGAATGGATCAGTAGTAATTCCAGAAGTCAGGCATACAAGGTGACATGCAGCGTGCGCCAGAGCAGCGCCCAGAACCGGAAATACACCATAAAAGTGGAGGTGCCCAAGGGTGCATGGCGCAGCTATCTCAATATGGAACTGACTATCCCCATCTTTGCGACCAACAGTGACTGCGAACTTATCGTGAAAGCTATGCAGGGGCTGCTTAAGGACGGTAACCCAATACCTTCAGCCATTGCCGCCAATTCCGGCTAA** |
| Nanoluc | **ATGGTCTTCACACTCGAAGATTTCGTTGGGGACTGGCGACAGACAGCCGGCTACAACCTGGACCAAGTCCTTGAACAGGGAGGTGTGTCCAGTTTGTTTCAGAATCTCGGGGTGTCCGTAACTCCGATCCAAAGGATTGTCCTGAGCGGTGAAAATGGGCTGAAGATCGACATCCATGTCATCATCCCGTATGAAGGTCTGAGCGGCGACCAAATGGGCCAGATCGAAAAAATTTTTAAGGTGGTGTACCCTGTGGATGATCATCACTTTAAGGTGATCCTGCACTATGGCACACTGGTAATCGACGGGGTTACGCCGAACATGATCGACTATTTCGGACGGCCGTATGAAGGCATCGCCGTGTTCGACGGCAAAAAGATCACTGTAACAGGGACCCTGTGGAACGGCAACAAAATTATCGACGAGCGCCTGATCAACCCCGACGGCTCCCTGCTGTTCCGAGTAACCATCAACGGAGTGACCGGCTGGCGGCTGTGCGAACGCATTCTGGCGTAA** |
| FiAsH | **ATGTGGGACTGCTGTGACGAATGCTGTAAGACGCGTGGAGGTGGCCATCACCATCATCACCATCACCACGGATAA** |
| EcorI | **ATGGGCCATCACCATCATCACCATCACCACGGAGGTGGCACTAGTGCTAGAAATGCAACAAACAAGTTACTGCACAAAGCTAAAAAATCGAAAAGCGACGAATTTTACACTCAGTATTGTGATATTGAGAACGAACTGCAATACTACAGAGAGCACTTCTCTGATAAGGTTGTTTATTGTAATTGCGATGATCCTAGAGTAAGCAATTTCTTTAAATATTTTGCAGTGAATTTTGATAATCTTGGCTTGAAAAAGTTAATAGCATCTTGCTATGTAGAGAATAAAGAAGGTTTTTCTAGTAGCGAAGCCGCGAAGAACGGATTTTACTATGAATATCATAAAGAAAATGGAAAGAAATTAGTTTTTGATGATATTAGTGTTTCTTCTTTCTGTGGCGATGGCGATTTTCGCAGTTCGGAGAGCATTGATCTGCTAAAAAAATCAGATATTGTTGTTACGAATCCTCCATTCTCGTTATTTAGAGAGTATCTTGATCAACTAATTAAGTATGATAAGAAATTCCTTATAATTGCTAATGTTAATTCAATAACATATAAAGAGGTGTTTAATCTAATTAAGGAAAATAAGATTTGGCTTGGGGTTCATCTCGGGAGAGGTGTTTCTGGATTTATTGTTCCAGAGCATTATGAATTATATGGTACTGAGGCGAGAATTGATTCTAATGGTAATAGAATTATCTCGCCAAACAACTGCTTATGGCTAACTAACCTAGATGTCTTTATTAGGCATAAAGACTTGCCTCTTACAAGAAAATATTTTGGGAATGAAAGTTCATATCCAAAATATGATAATTATGATGCTATAAATGTAAACAAAACAAAGGATATTCCATTAGATTACAATGGGGTTATGGGGGTTCCTATCACATTCTTGCATAAGTTTAACCCTGAGCAATTTGAGTTAATAAAATTTAGAAAGGGTGTTGATGAAAAAGATTTGTCTATAAATGGTAAATGCCCTTATTTCAGAATTTTGATAAAAAACAAACGATTACAAAAG** |
| Wheat Germ extract | [promoter]CAGTGAAGATTGACCATCTCACAAAAGCTGTTACGTGCTTGTAACACACTACACACTCGTTTTGTATTCGAGAAGTAGTTGCAACAACGGTCCCCTTATTGCCTGACAAGCTGAGGGCCACCCTTCTATCCCCACCGCGCGATCGCCATGGAATAAGTAAGGAATCCAC[GFP]TGTTTAAACGAATTCGAGCTCGGTACCAGTGAAGACAACACCACTAGCACAAATCGGATCCTGGGAAACAGGCAGAACTTCGGTTCATAAGCTCGGGTAGGCTGTCAACCTACCGCCGTATCGTATTGTGTTTGTCTAGATACGTATCGCGAGTCGACCTGCAGGCATGCAAGCTGATCCGGCTGCTAACAAAGCCCGAAAGGAAGCTGAGTTGGCTGCTGCCACCGCTGAGCAATAACTAGCATAACCCCTTGGGGCCTCTAAACGGGTCTTGAGGGGTTTTTTG |
| Rabbit reticulocyte extract expression cassette | [promoter]AATACAAGCTACTTGTTCTTTTTGCACTCGAGAATTCACGCGTGGTACCTCTAGAGTCGACCCATC[GFP]GATGGGCGGCCGCAAAAAAAAAAAAAAAAAAAAAAAAAAAAACCTAGCATAACCCCTTGGGGCCTCTAAACGGGTCTTGAGGGGTTTTTT |
| TnT extract expression cassette | [promoter]AGTATTTTTATTCTTTCGTAAAAAAATTAGAAAAATAAAATATAAAGCGATCGCCATGGAATAAGTAAGGAATCCAC[GFP]GTTTAAACGAATTCGGGCTCGGTACCCGGGGATCCTCTAGAGTCGACCTGCAGGCATGCAAGCTGATCCGGCTGCTAACAAAGCCCGAAAGGAAGCTGAGTTGGCTGCTGCCACCGCTGAGCAATAACTAGCATAACCTAGAGTCGGGCGGCTGTAAAACACGATACATTGTTATTAGTACATTTATTAAGCGCTAGATTCTGTGCGTTGTTGATTTACAGACAATTGTTGTACGTATTTTAATAATTCATTAAATTTATAATCTTTAGGGTGGTATGTTAGAGCGAAAATCAAATGATTTTCAGCGTCTTTATATCTGAATTTAAATATTAAATCCTCAATAGATTTGTAAAATAGGTTTCGATTAGTTTCAAACAAGGGTTGTTTTTCCGAACCGATGGCTGGACTATCTAATGGATTTTCGCTCAACGCCACAAAACTTGCCAAATCTTGTAGCAGCAATCTAGCTTTGTCGATATTCGTTTGTGTTTTGTTTTGTAATAAAGGTTCGACGTCGTTCAAAATATTATCCGCAAAAAAAAAAAAAAAAAAAAAAAAAAAAAACTAGCATAACCCCTTGGGGCCTCTAAACGGGTCTTGAGGGGTTTTTTG |
| Leishmania extract expression cassette | [promoter]GACATCTTAAGTTTATTTTATTTTATTTTATTTTATTTTATTTTATTTTATTTTATTTTATTTTATTTTATTTAACC[GFP]GCGGCCGCCCTCCTCCTCCTTTCTTGTTCCTTTCACGTCGCCTTCTCGGTTGTAGCTGGCAGACGACGAGTCTTACTTTTACGTGTACTTCTCTATAGATGATGTATGATCTCTCTGCATGCGTGTTCGTGCATGTGTCCGTGTGTTGGGTACGCGT |
|  |  |
